# Adsorption on a Spherical Colloidal Particle from a Mixture of Nanoparticles with Competing Interactions

**DOI:** 10.3390/molecules29133170

**Published:** 2024-07-03

**Authors:** Marek Litniewski, Wojciech T. Góźdź, Alina Ciach

**Affiliations:** Institute of Physical Chemistry, Polish Academy of Sciences, 01-224 Warsaw, Poland; mlitniewski@ichf.edu.pl (M.L.); wtg@ichf.edu.pl (W.T.G.)

**Keywords:** colloidal self-assembly, spontaneous pattern formation on a sphere, inhomogeneous mixtures, molecular dynamics simulations, self-assembled stripes, adsorption on curved surfaces

## Abstract

Adsorption of nanoparticles on a spherical colloidal particle is studied by molecular dynamics simulations. We consider a generic model for a mixture of nanoparticles with energetically favored self-assembly into alternating layers of the two components. When both components are attracted to the colloidal particle, the adsorbed nanoparticles self-assemble either into alternating parallel tori and clusters at the two poles of the colloidal particle, or into alternating spirals wrapped around the spherical surface. The long-lived metastable states obtained in simulations follow from the spherical shape of the adsorbing surface and the requirement that the neighboring chains of the nanoparticles are composed of different components. A geometrical construction leading to all such patterns is presented. When the second component particles are repelled from the colloidal particle and the attraction of the first component is strong, the attracted particles form a monolayer at the surface of the colloidal particle that screens the repulsion of the second component. The subsequent adsorbed alternating spherical layers of the two components form together a thick shell. This structure leads to the adsorption that is larger than in the case of the same attraction of the two components to the colloidal particle.

## 1. Introduction

Self-assembly into different aggregates is ubiquitous in biological and soft-matter systems that typically contain amphiphilic molecules and/or charged macromolecules or nanoparticles. In mixtures with water or other solvents, amphiphilic molecules self-assemble into aggregates such as spherical or cylindrical micelles, networks or bilayers. On the other hand, charged particles with solvent-induced short-range attraction can self-assemble into clusters with spherical or cylindrical shape, networks or layers [1,2,3,4,5,6,7,8,9,10,11,12].

Micells and clusters of similar shapes can be packed in a similar way, and the phase diagrams in these systems are strikingly similar [13], despite different chemical nature of the self-assembling particles or molecules, and different solvents. Similarity of the phase diagrams in many different systems indicates that all microscopc interactions average to effective interactions between the particles or molecules that in different systems have similar features, and govern the self-assembly and distribution of the assemblies in space. Thus, to determine the stable or metastable distribution of the assemblies, one assumes that the solvent induces effective interactions between the particles, and the solvent molecules are taken into account only indirectly. This type of mesoscopic description turned out to be successful for colloidal particles and globular proteins, and is a standard method in studies of structural and thermodynmic properties of solutions of particles much larger than the solvent molecules [14,15,16]. Solvent-induced effective attraction between particles may have different origin. For example, addition of nonadsorbing polymers with small radius of gyration to the solvent leads to the so called depletion attraction between large particles present in the solvent [1,2]. In another example, concentration fluctuations in near-critical mixture lead to the so called thermodynamic Casimir potential between objects preferentially adsorbing one component of the mixture [17].

The effective attraction induced by the solvent competes with the electrostatic repulsion between charged particles, regrdless of its origin. The competition between the short-range attraction and long-range repulsion is the key factor leading to the self-assembly of charged particles in complex solvents. The same sequence of ordered patterns was obtained for different shapes of the attractive and repulsive parts of the effective potential, although the size of the assemblies in different systems can be significantly different. Based on the knowledge about self-assembly in natural systems, one can design materials with desired patterns on different length scales. Thus, fundamental knowledge about properties common for many self-assembling systems is important.

The self-assembly of the particles or globular proteins into clusters in a dilute solution that is in contact with attractive wall can significantly influence the adsorption [18]. Once a layer of particles with short-range attraction long-range repulsion (SALR) is adsorbed, it forms a repulsive barrier and formation of the next layer upon increasing chemical potential competes with self-assembly of the monomers into clusters in the bulk. Because of the type of the interactions, the adsorbed particles self-assemble into clusters or stripes, and form a regular pattern on the surface rather than a dense layer [19,20].

Recently, mixtures of two types of self-assembling particles attract increasing attention [16,21,22,23,24,25,26,27], because the aggregates and patterns expected in such mixtures may find numerous practical applications depending on the properties of the nanoparticles. In this work we focus on a mixture inspired by oppositely charged hydrophilic and hydrophobic nanoparticles suspended in water-oil mixture that is close to the miscibility critical point. In this system, like particles interact with the SALR potential if the screening length is larger than the range of the thermodynamic Casimir potential induced by concentration fluctuations in the binary solvent [17,28,29,30,31,32,33,34,35]. The cross-interaction, however, is repulsive at short and attractive at large distances. For such interactions, alternating layers of the two types of particles are energetically favoured. For equal numbers of particles of the two components, the phase diagram obtained in theory and in molecular dynamics (MD) simulations is relatively simple—a dilute solution coexists with a dense phase of alternating layers of the two types of particles [23,24].

The presence of the second-component nanoparticles has a very significant effect on the adsorption on a flat surface [36]. Because the nanoparticles of the second component are attracted to the first-component nanoparticles at the distances where like nanoparticles are repelled, the adsorption in this model mixture is significantly larger than in the one-component SALR system. The alternating layers of the two components can be perpendicular or parallel to the adsorbing surface, depending on the interactions between the surface and the nanoparticles.

It is not obvious how the curvature of the surface influences the self-assembly of the adsorbed nanoparticles, and how the amount of adsorbed nanoparticles depends on the pattern in the adsorbed shell. In the case of the one-component SALR model, the effect of curvature of the surface on self-assembled patterns was studied in [37]. To answer these questions for self-assembling mixtures, we focus on a binary mixture of nanoparticles with the competing interactions discussed above, and on adsorption of these nanoparticles on a spherical colloidal particle. We consider interactions between the colloidal particle and the nanoparticles either attractive for both components, or attractive for the first one and repulsive for the second one. To model the adsorption of nanoparticles with the diameter σ∼10 nm at the colloidal particle with the radius $R∼10^{2}-10^{3}$ nm, we consider 5≤R≤40 in units of the diameter of the nanoparticles. In our MD simulations, we investigate the structure in the adsorbed layer, and calculate density profiles and the adsorption of both components for different curvatures and interactions between the colloidal particle and the nanoparticles.

In Section 2, we define the model and the simulation method. In Section 3, we present the density profiles and the structure of the adsorbed layers, as well as the adsorption and the difference between the adsorption of the first and the second component for different strengths of the interactions between the colloidal particle and the nanoparticles, and different *R*. For strong attraction between the nanoparticles and the colloidal particle, the surface of the latter can be covered by a dense layer of the nanoparticles. Because alternating layers of the two components are energetically favored, regions occupied by different particles are enclosed by mathematical surfaces of flexible tubes. In Section 4, we present all geometrically possible patterns formed by two types of flexible tubes densely wrapped around a surface of a sphere such that the neighboring tubes are of different types. We compare the obtained patterns with our simulation results. The final section contains our conclusions.

## 2. Model and Simulation Method

In our generic model, only solute particles are directly taken into account, and the solvent molecules lead to effective interactions between the particles. We assume the same potential as in the studies of the bulk phase diagram [23] and in the studies of adsorption on a flat wall [36]. For the interaction between like particles we assume:

(1) $$u_{ii}\left(r\right)=\frac{6\epsilon }{r^{12}}-\frac{6\epsilon }{r^{6}}+1.8\frac{e^{-r/2}}{r},$$

where i=1,2, and the cross interaction has the form

(2) $$u_{12}\left(r\right)=\frac{6\epsilon }{r^{12}}+\frac{6\epsilon }{r^{6}}-1.8\frac{e^{-r/2}}{r}.$$

*r* is the distance between the particles in units of the particle diameter σ. We choose σ for the unit length in the rest of the article. The potential is truncated at $r=r_{c}=6.75$.

We consider dilute mixture of particles with the above interactions, and adsorption of these particles on spherical surfaces. Two types of interactions between the adsorbing surface and the particles are distinguished. In the first case, both components are attracted to the sphere with the potential

(3) $$V_{attr}\left(r\right)=2\gamma_{attr}\left[{\left(\frac{1}{r+1-R}\right)}^{12}-{\left(\frac{1}{r+1-R}\right)}^{6}\right].$$

We consider attractions with the strengths $\gamma_{attr}=2,4,6,8,12$. In the second case, particles of the first component are attracted to the adsorbing sphere with the potential (3), but the particles of the second component are repelled from this sphere with the potential

(4) $$V_{rep}\left(r\right)=0.5{\left(\frac{1}{r+1-R}\right)}^{12}.$$

**Figure 1:**
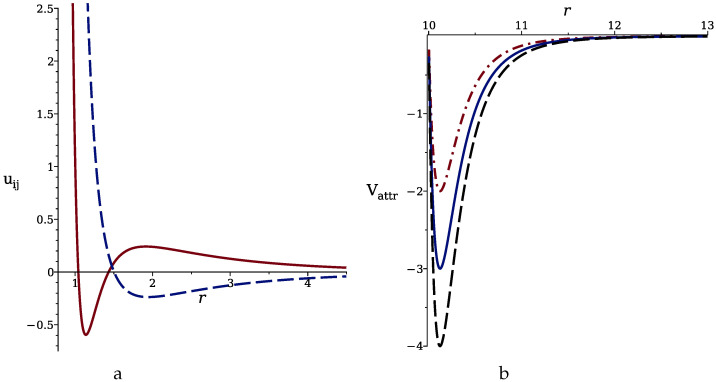
(**a**) Effective interaction potential between like particles (Equation (1), solid line) and cross-interaction (Equation (2), dashed line) for ϵ=1. (**b**) Attractive potential (3) between the nanoparticles and the adsorbing sphere with radius R=10. Dashed, solid and dash-dotted lines correspond to $\gamma_{attr}=8,6,4$, respectively. *r* is in units of the particle diameter.

In the above formulas, *r* is the distance between the centers of the particle and the adsorbing sphere with the radius *R*. $u_{ij}$ and $V_{attr}$ are shown in Figure 1.

The MD simulations were performed in a 3D box with the edges $L_{x}=L_{y}=L_{z}=L=396.5$. Periodic boundary conditions were assumed in each direction. The number of particles of the first component, $N_{1}$, was equal to the number of particles of the second one, $N_{2}$, and was adjusted to the dimensionless gas density $\rho_{g}=\left(N_{1}+N_{2}\right)/L^{3}=0.0023$. The simulations were performed at the temperature $\bar{T}=k_{B}T/\epsilon =0.25$, where $k_{B}$ is the Boltzmann constant. The temperature was kept constant by scaling the particle velocities in a way described in detail in Reference [23]. The density $\rho_{g}=0.0023$ was chosen because it is slightly smaller than the gas density $\rho_{g}\approx 0.0027$ at the gas-crystal coexistence at $\bar{T}=0.25$ for which the simulations were performed [23]. We choose these thermodynamic parameters because for the interactions (1) and (2) the crystal coexisting with the gas at $\bar{T}=0.25$ is composed of alternating bilayers of the two components [23], and this structure of the ordered phase may be reflected in the film adsorbed at the spherical surface.

To study the adsorption, the adsorbing impenetrable sphere with the radius 5≤R≤40 was introduced at the center of the box and kept at the fixed position. During the simulations, the particles were adsorbed at the sphere. To keep the gas density away from the adsorbing sphere constant, new particles were added by applying the procedure described in detail in ref. [36]. The conditions for adding the particles and the process itself were applied for r>150.

We determined the density profiles of each component as functions of the distance from the center of the adsorbing sphere, as well as the adsorptions: the total one, Γ, and the selective one, $\Gamma_{c}$, defined as follows:

(5) $$\Gamma =\frac{1}{R^{2}}\int_{R}^{\infty }\left(\rho_{1}\left(r\right)+\rho_{2}\left(r\right)-\rho_{g}\right)r^{2}dr$$

and

(6) $$\Gamma_{c}=\frac{1}{R^{2}}\int_{R}^{\infty }\left(\rho_{1}\left(r\right)-\rho_{2}\left(r\right)\right)r^{2}dr.$$

The total number of particles adsorbed at the sphere is $4\pi R^{2}\Gamma$, while $4\pi R^{2}\Gamma_{c}$ is the difference between the number of the first- and the second component particles adsorbed at the sphere.

## 3. Results

### 3.1. Adsorption at the Sphere Attracting Both Components

**Figure 2:**
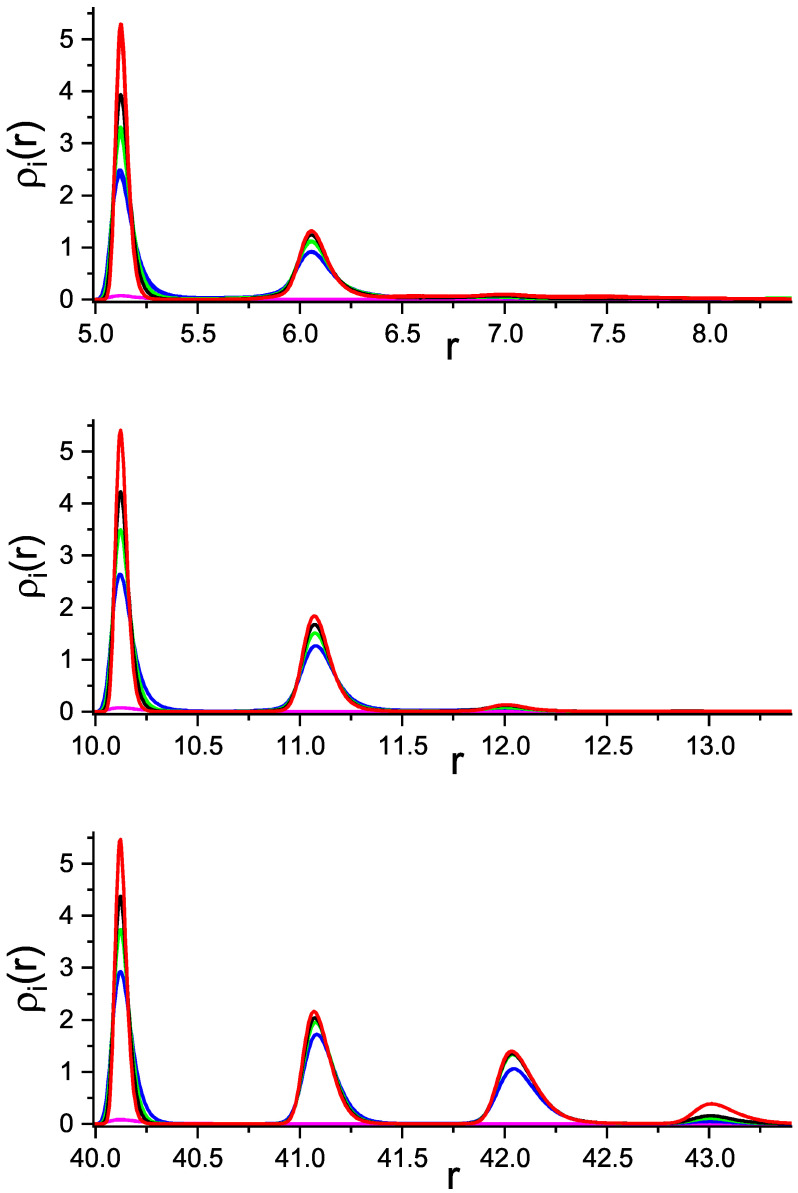
Density profiles $\rho_{1}\left(r\right)=\rho_{2}\left(r\right)$ of the two components attracted to the sphere with the potential (3), as functions of the distance *r* from the center of the adsorbing sphere with the radius R=5 (top), R=10 (central) and R=40 (bottom panel). Pink, blue, green, black and red lines (from bottom to top) correspond to $\gamma_{attr}=2,4,6,8,12$, respectively.

We first present results for the sphere attracting both components with the same strength (see Equation (3)). In this case $\rho_{1}\left(r\right)=\rho_{2}\left(r\right)$, and the density profiles are shown in Figure 2. Rather narrow maxima separated by a distance equal to the particle diameter indicate formation of well localized smooth shells with equal number of particles of both components in each shell. In Figure 2, the profiles are shown for R=5, R=10 and R=40. We can see that the number of adsorbed layers as well as the number of particles in each layer increase with increasing *R* and $\gamma_{attr}$ for the attraction strengths $\gamma_{attr}\geq 4$. $\gamma_{attr}=2$ is too weak to attract noticable number of particles.

**Figure 3:**
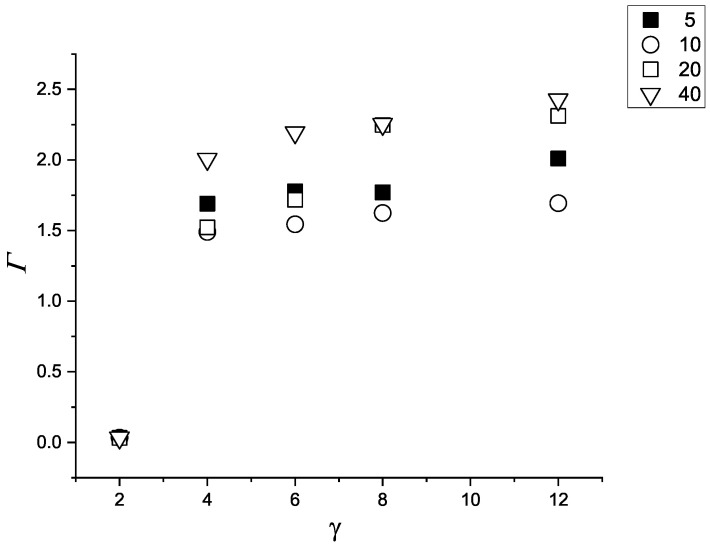
Adsorption Γ at the sphere attracting both components with the potential (3) and attraction strengths $\gamma_{attr}=2,4,6,8,12$, as a function of $\gamma_{attr}$. Black filled squares, open circles, open squares and triangles represent R=5,10,20,40, respectively.

The adsorption in this case is shown in Figure 3.

For $\gamma_{attr}\geq 4$, the adsorption increases only a little with increasing $\gamma_{attr}$ for all values of *R*. For $\gamma_{attr}=4$, we have 1.5≤Γ≤2 and for $\gamma_{attr}=12$, 1.7≤Γ≤2.4 for 5≤R≤40. Because of larger area $4\pi R^{2}$ of the surface of the sphere, the number of adsorbed particle is of course larger at the larger sphere. The adsorption at the smallest sphere is a bit larger than for R=10, because the relative increase of the area of the monolayer shifted by 1 (the particle diameter) from the surface of the adsorbig sphere is $2/R+1/R^{2}$, i.e., it is larger for smaller *R*. The effect is seen also for R=20 for $\gamma_{attr}\leq 6$. However, when *R* and $\gamma_{attr}$ increase, the number of adsorbed layers increases as well (Figure 2) leading to increasing adsorption, and the resulting adsorption depends on the competition between these two effects. Due to the symmetry, $\Gamma_{c}=0$ in this case.

**Figure 4:**
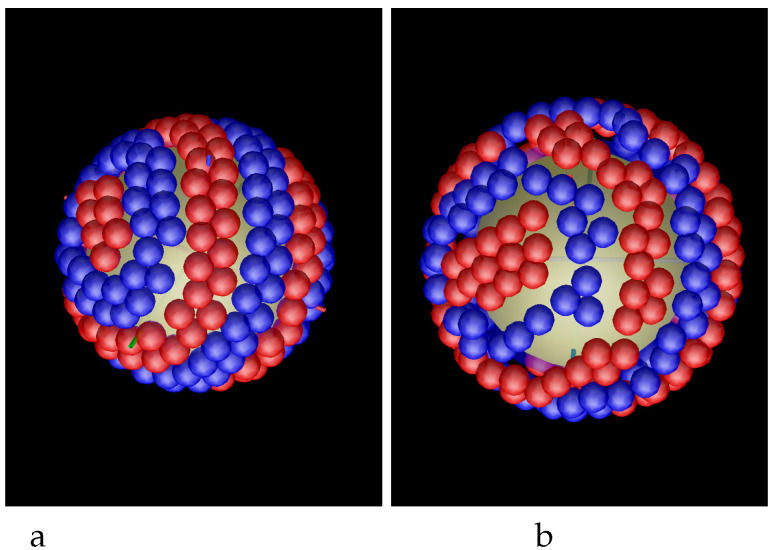
Snaphots showing the first (**a**) and the second (**b**) layers of adsorbed particles at the sphere with radius R=5 attracting both components with the potential (3) and $\gamma_{attr}=4$. The snapshots show the patterns rotated with respect to each other, but in the final configuration the layers in the second monolayer are located on top of the layers of the same color in the inner monolayer. Red and blue spheres represent particles of the first and the second component, respectively.

**Figure 5:**
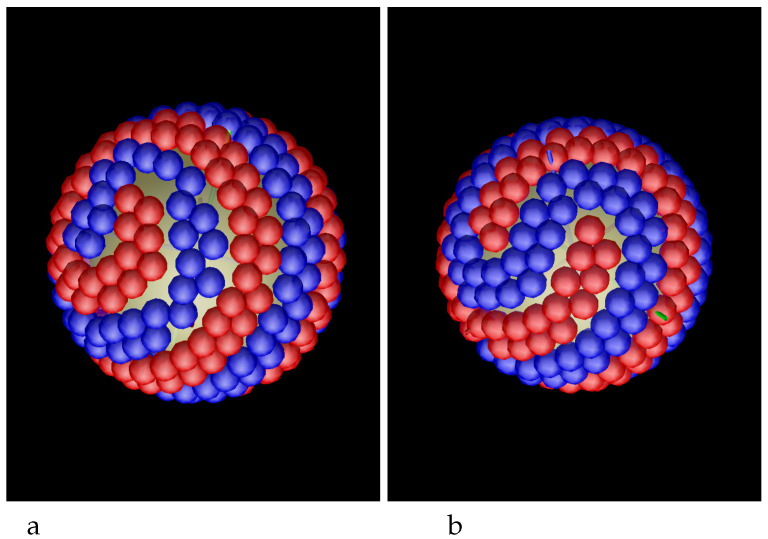
Snaphots showing the first layers of particles adsorbed at the sphere attracting both components with the potential (3). (**a**) R=5.5 and $\gamma_{attr}=4$. (**b**) R=5 and $\gamma_{attr}=8$. Note the more densely packed particles in the right panel because of larger attraction to the sphere. Red and blue spheres represent particles of the first and the second component, respectively.

**Figure 6:**
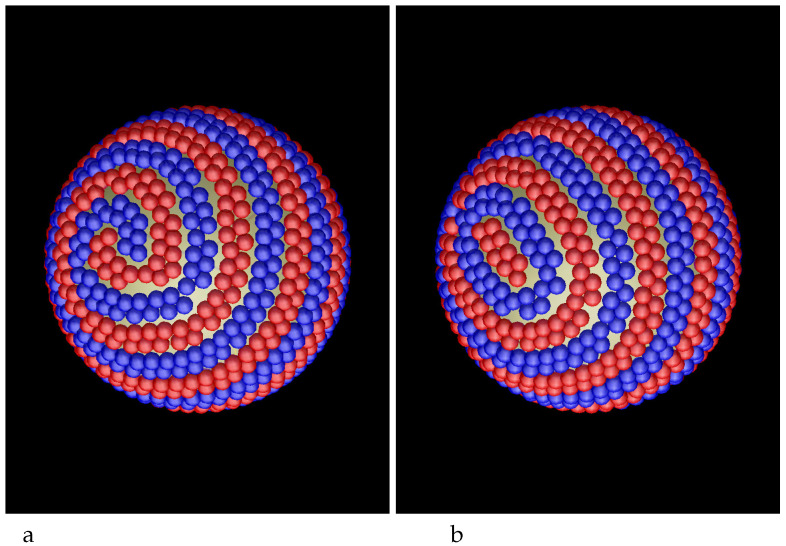
Snaphots showing the first layers of particles adsorbed at the sphere attracting both components with the potential (3) with $\gamma_{attr}=4$ for R=10. The structures shown in panels (**a**,**b**) were obtained by MD simulations from different initial conditions. Red and blue spheres represent particles of the first and the second component, respectively.

In the case of the attractive sphere, the density profiles do not give sufficient information about the structure of the adsobed film. Because of the competing interactions, the two components should microsegregate, and the segregation takes place inside the adsorbed spherical layers of the particles, not between these layers. The structure of the first and the second monolayer adsorbed at the sphere is shown in the snapshots presented in Figure 4 for R=5 and $\gamma_{attr}=4$. We see alternating toruses formed by bilayers of particles of the first and the second component, and clusters at the poles. The pattern is repeated in the second monolayer, but the particles in the second layer are more loosely packed because the area of the surface at the distance R+1 is larger than the area of the surface at the distance *R* from the center of the adsorbing sphere. In some cases, spirals instead of tori appear, as can be seen in Figure 5 for R=5.5 and $\gamma_{attr}=4$ and for R=5 and $\gamma_{attr}=8$ and in Figure 6 for R=10 and $\gamma_{attr}=4$. Due to low temperature, the obtained pattern corresponds either to stable or to long-lived metastable state, therefore different patterns can be obtained from different initial conditions. The patterns that can appear at the attractive sphere will be discussed in more detail in Section 4.

### 3.2. Adsorption at the Selective Sphere

**Figure 7:**
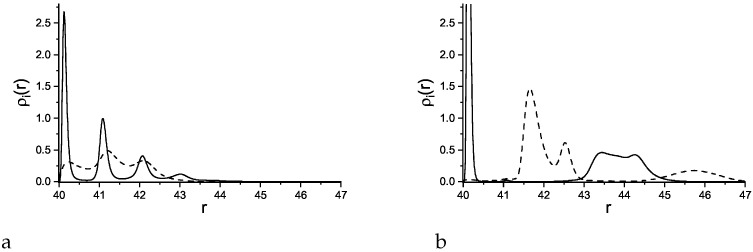
Density profiles of the first, attracted component (solid lines) and the second, repelled component (dashed lines) as functions of the distance *r* from the center of the adsorbing sphere with the radius R=40. Attractive and repulsive interactions are given in Equations (3) and (4), respectively. The profiles are shown for $\gamma_{attr}=4$ (**a**) and for $\gamma_{attr}=6$ (**b**). The profiles for $\gamma_{attr}>6$ are qualitatively identical to that in the right panel.

**Figure 8:**
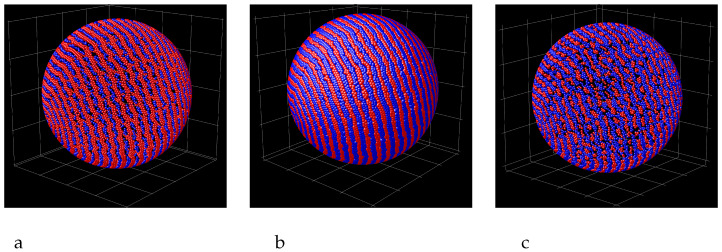
Snaphots showing the layers of particles adsorbed at the sphere attracting the first component (red spheres) with the potential (3) and $\gamma_{attr}=4$ and repelling the second component (blue spheres) with the potential (4) for R=40. (**a**) the first, (**b**) the second and (**c**) the third monolayer.

The density profiles in this case are significantly different from the profiles in the case of a nonselective surface. For small curvature of the sphere, we expect similar results as for adsorption at a planar wall. Indeed, for R=40 we obtain density profiles very similar to the profiles found in Ref. [36] for a planar wall for all the considered strengths of the wall-particle attractions (see Equation (3)). For $\gamma_{attr}=4$, the attracted particles form smooth monolayers as shown by rather narrow peaks of $\rho_{1}\left(r\right)$ in Figure 7, left panel. The maxima of $\rho_{2}\left(r\right)$ are shifted to larger distances from the adsorbing sphere and are much broader. Despite these differences, the patterns in the adsorbed layers (Figure 8) are similar to those discussed for the nonselective surface.

**Figure 9:**
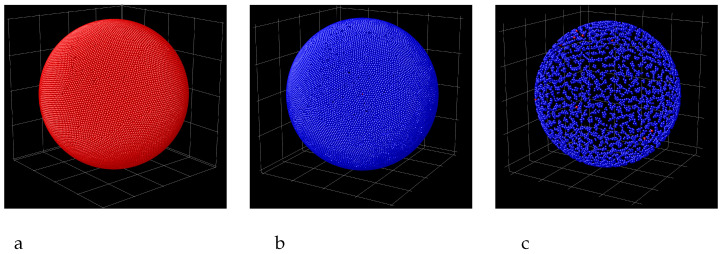
Snaphots showing the layers of particles adsorbed at the sphere attracting the first component (red spheres) with the potential (3) and $\gamma_{attr}=8$ and repelling the second component (blue spheres) with the potential (4) for R=40. (**a**) the first-component monolayer adsorbed at the sphere (the first maximum in Figure 7b). (**b**) the inner monolayer of the second-component bilayer separated from the first-component monolayer by the empty zone (the second maximum in Figure 7b). (**c**) the outer monolayer of the second-component bilayer (the third maximum in Figure 7b).

For $\gamma_{attr}\geq 6$, however, a significant structural change takes place. While for $\gamma_{attr}\leq 4$ the microphase separation occurs inside the adsorbed monolayers, for $\gamma_{attr}\geq 6$ the separation takes place between the monolayers, as clearly indicated by the shift of the maxima of the density of the second component with respect to the maxima of the density of the first component (Figure 7, right panel). The patterns for $\gamma_{attr}=8$ and R=40 are shown in Figure 9.

The first-component compact monolyer covering the sphere is separted by an empty layer of the thickness equal to the particle diameter from the following layer of the particles repelled from the wall. The subsequent adsorbed layers are patterned and porous, as for the adsorption on a flat surface [36].

**Figure 10:**
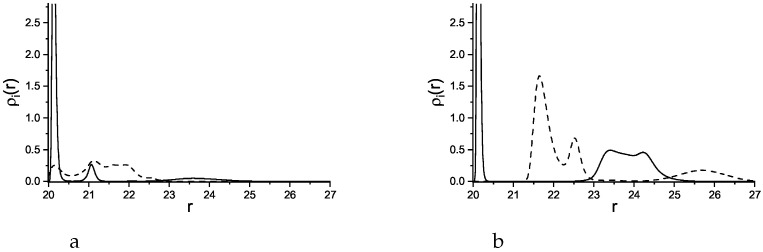
Density profiles of the first, attracted component (solid lines) and the second, repelled component (dashed lines) as functions of the distance *r* from the center of the adsorbing sphere with the radius R=20. Attractive and repulsive interactions are given in Equations (3) and (4), respectively. The profiles are shown for $\gamma_{attr}=6$ (**a**) and for $\gamma_{attr}=8$ (**b**).

The density profiles for R=20 are shown in Figure 10 for the strengths of the first-component attraction to the sphere $\gamma_{attr}=6$ and $\gamma_{attr}=8$, corresponding to two different structures of the adsorbed film. For $\gamma_{attr}\geq 8$, the density profiles are very similar to the profiles for R=40 and $\gamma_{attr}\geq 6$, indicating structure of the adsorbed film similar to the one shown in Figure 9. For $\gamma_{attr}\leq 6$, the adsorbed film is thinner and less dense. The first component particles form a bilayer at the surface, with the inner monolayer dense, and the outer one dilute. The repelled component forms a dilute cloud that spreads from the surface of the sphere up to the third layer.

**Figure 11:**
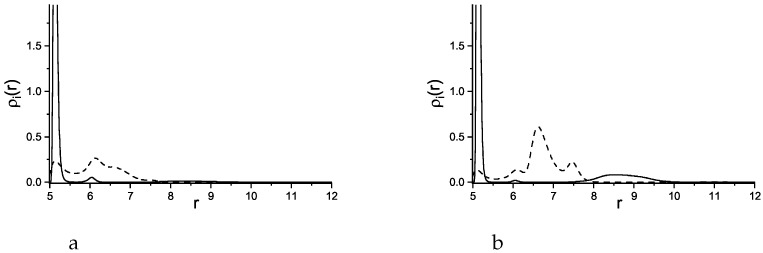
Density profiles of the first, attracted component (solid lines) and the second, repelled component (dashed lines) as functions of the distance *r* from the center of the adsorbing sphere with the radius R=5. The profiles are shown for $\gamma_{attr}=6$ (**a**) and for $\gamma_{attr}=8$ (**b**).

For the smallest sphere with R=5 (Figure 11), only a monolayer of the first-component particles is adsorbed for $\gamma_{attr}<6$, and for $\gamma_{attr}=6$ (Figure 11a), the first-component monolayer is surrounded by a thick but low-density cloud of the second component. For $\gamma_{attr}\geq 8$, the attracted component forms a smooth monolayer at the surface that is followed by a low density, disordered bilayer of the second component and a diffuse cloud of the attracted particles (Figure 11b). Despite much less ordered structure, there still exists a structural change resembling the one described for larger *R*, with almost coinciding maxima of the two components for $\gamma_{attr}\leq 6$, and well separated maxima (alternating spherical layers of the two components) for $\gamma_{attr}\geq 8$.

**Figure 12:**
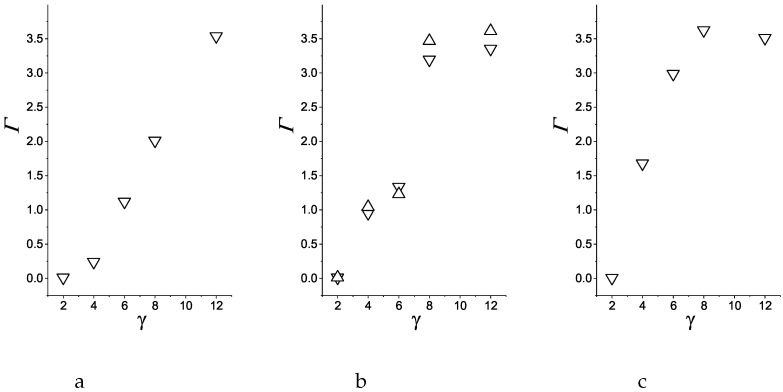
Adsorption Γ for the surface attracting the first component with (3) and attraction strengths $\gamma_{attr}=2,4,6,8,12$, and repelling the second component with the potential (4). (**a**) R=5, (**b**) triangles down: R=10, (**b**) triangless up: R=20 and (**c**) R=40.

In Figure 12 we show the adsorption as a function of the strength of the attraction for R=5 (Figure 12, left), for R=10,20 (Figure 12 center) and R=40 (Figure 12 right). As already mentioned, for $\gamma_{attr}=2$, the adsorption is negligible in all cases. As seen in Figure 12, for R=5 the adsorption increases almost linearly for $\gamma_{attr}$ increasing from 4 to 12. For larger *R*, however, the adsorption increases rapidly between $\gamma_{attr}=6$ and $\gamma_{attr}=8$, because of the significant structural change in the adsorbed film for $6<\gamma_{attr}<8$ (compare Figure 8 and Figure 9). For very strong attraction of the first component, Γ takes almost the same value for R≥10 that is also nearly the same for $\gamma_{attr}=8$ and $\gamma_{attr}=12$. Finally, we should note that the adsorption at the sphere with very large radius R=40 is significantly larger from the adsorption for R≤20, except from very strong attraction of the first component. The largest difference is for $\gamma_{attr}=6$. This is due to formation of compact layers around the sphere with R=40, and rather porous disordered patterns for R≤20 when the attraction to the sphere is not very strong.

Note the significantly smaller adsorption at the sphere attracting both components with the potential (3) and large attraction strength $\gamma_{attr}\geq 8$, than at the sphere attacting the first component with the same strength, and repelling the second one with the potential (4). For $\gamma_{attr}=8$, we have Γ∼2 or Γ∼3 for the sphere attracting both components or for the selective one, respectively. It is a consequence of the structural reorganization at the selective sphere and formation of alternating spherical shells of the two components around the particle. For weaker attraction of the first component to the selective surface the adsorption at the selective surface is smaller.

**Figure 13:**
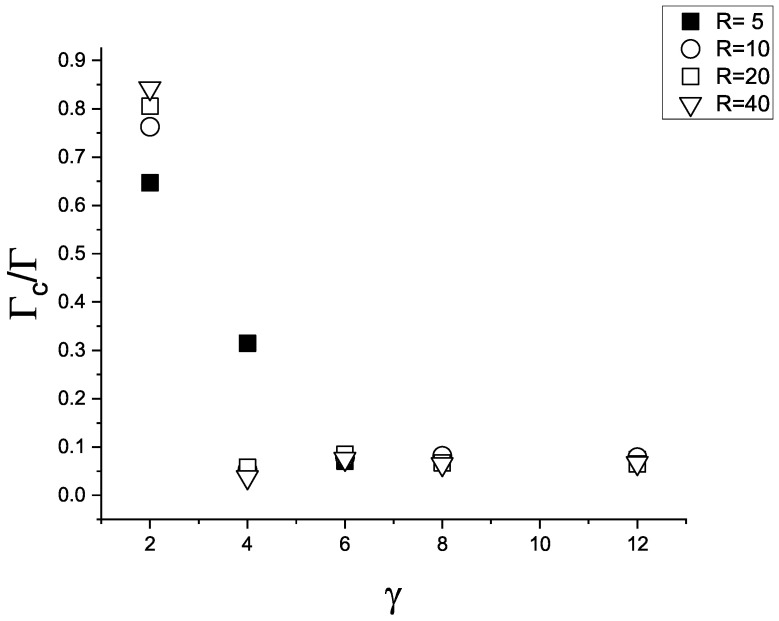
The ratio of the selective and total adsorption $\Gamma_{c}/\Gamma$ for the surface attracting the first component with (3) and attraction strengths $\gamma_{attr}=2,4,6,8,12$, and repelling the second component with the potential (4). Black filled squares, open circles, open squares and triangles represent R=5,10,20,40, respectively.

Since we study the adsorption at a selective surface, we expect $\Gamma_{c}>0$. As shown in Figure 13, $\Gamma_{c}$ is quite small, indicating very similar number of particles of the attracted and the repelled component in the adsorbed film. In addition, the ratio $\Gamma_{c}/\Gamma$ is almost independent of $\gamma_{attr}$ and of *R*. Only for $\gamma_{attr}=2$, and $\gamma_{attr}=4$ for R=5, the adsorbed film is rich in the first component. In these cases, however, Γ is quite small. If a substantial amount of particles is adsorbed at the sphere, the excess of the attracted particles over the repelled ones is very small.

## 4. Discussion

In this section we focus on the sphere attracting both components with the same strength. For strong attraction and relatively low temperature, the adsorbed particles are rather closely packed. In order to pack closely a binary mixture of spherical molecules interacting with the potential given by Equations (1) and (2), we can assume that the nanoparticles of one kind form chains in contact with the chains of the nanoparticles of the other kind. Such an assumption is supported by the results of the simulations, as shown in Figure 4, Figure 5 and Figure 6. We can approximate the boundaries of the regions occupied by the nanoparticles by flexible tubes of two colors enclosing the regions rich in the nanoparticles of the two types. We have constructed possible arrangements of the molecules on a sphere in analogy to the arrangement of the optimally packed rope of a given thickness on a surface of a sphere [38,39].

**Figure 14:**
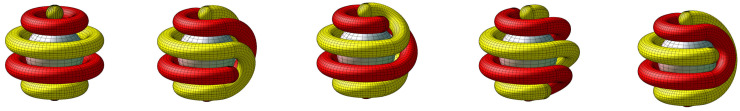
Geometrically possible arrangements of two ropes of different color densely wrapped around a spherical surface, with ropes of different color located next to each other. The left configuration shows 4 alternating tori parallel to the equator, and spheres of different color located at the two poles. The remaining patterns are obtained from this parent structure by the procedure described in the text.

**Figure 15:**
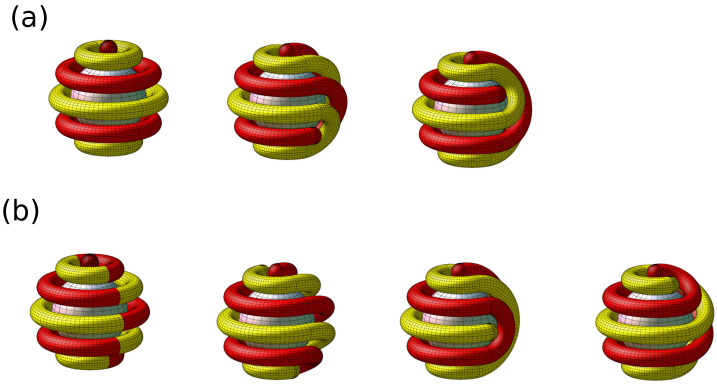
Geometrically possible arrangements of two ropes of different color densely wrapped around the spherical surface, with ropes of different color located next to each other. The left configuration in panel (**a**) shows 5 alternating tori parallel to the equator, and spheres of the same color located at the two poles. The remaining patterns in (**a**) are obtained from this parent structure by the procedure described in the text. The left configuration in panel (**b**) shows the two joint hemispheres with the connected half-tori of different color. The remaining patters in (**b**) are obtained from the above structure by rotation of one of the hemispheres such that the half-tori of the same color are smoothly connected.

The difference between the previous case concerning the one-component system of particles with the SALR potential confined in a spherical shell and the current binary mixture is such that now we consider the pieces of the rope with two colors under the restriction that the ropes of the same color have to be separated by the rope of different color. In order to construct possible configurations, we start with a sphere covered by tori and two small spheres at the poles, keeping the radii of the small spheres and tori the same. Next, we cut such a geometric object by a plane containing the line that joins the centers of the small spheres at the poles of the large sphere. We obtain two large hemispheres covered with half tori. New configurations are obtained by rotating one of the hemispheres in such a way to be able to connect smoothly the pieces of the tori of the same color as shown in Figure 14 and Figure 15a.

We can distinguish even and odd number of tori on the adsorbing sphere, with the clusters of different components on the poles in the first case, and clusters composed of particles of the same component in the second one. For even number of tori, the above precedure leads to all possible spiral patterns. For odd numbers of tori, however, we can obtain more structures by changing the color of all the half-tori and small spheres at one of the two hemispheres and performing the rotation, but with the rotation angle two times smaller than in the previous construction, as shown in Figure 15b.

In our simulations, the sphere with R=5 was covered by 5 tori (Figure 4). Note that the patterns obtained in simulations and shown in Figure 5, left and right panel, agree with the last figure in the bottom row and the last figure in the top row in Figure 15, respectively. Similarly, the pattern obtained for R=10 and shown in Figure 6 has the structure obtained by our mathematical procedure and shown in the last cartoon in the bottom row in Figure 15.

The structure of the adsorbed layer corresponds either to the equilibrium state, or to a long-lived metastable state. For the interactions (1) and (2), the energy is minimized when alternating bilayers of the two components are formed. As can be seen from the snapshots in Figure 4 and Figure 5, the energy is low for both the tori and the spirals, but the grand potentials in the two cases can be somewhat different. When the system is trapped in a deep energy minimum corresponding to an ordered pattern, a collective movement of the majority of the particles is necessary for changing the pattern to the one corresponding to the global minimum of the grand thermodynamic potential. Because of the glassy-like feature, different initial conditions of the particles in the gas can lead to different long-lived patterns. The limitation for all possible patterns that may occur at relatively low temperature at strongly attractive surface is of both, geometrical and energetic nature, as shown by our MD simulations. Figure 14 and Figure 15 show the geometrically possible patterns in the mixture with energetically favored alternating layers of the two components at the sphere that can contain 4 and 5 densely packed tori, respectively.

Remarkably, we obtained the possible patters in the two-component film adsorbed at an attractive sphere using the same mathematical procedure as for the patterns self-assembled by the particles with the SALR potential leading to assemblies as thick as 5 particle diameters, confined inside a spherical shell with hard walls. Perhaps counterintuitively, the number of possible patterns in the microsegregating mixture is significantly smaller than in the one-component system with the SALR interactions [38,39]. This is because of the restriction that neighboring tori in the parent structure must contain different components, and only the half-tori enclosing the same component can be connected (Figure 14 and Figure 15).

The simulations were performed for the interactions between like particles and the cross-interaction of the form (1) and (2) respectively, leading to alternating bilayers of the particles in the bulk. The thickness of the alternating layers of the particles in the dense phase in the bulk can be different for different width and depth of the attractive well and different range of the repulsive barrier between like particles [23]. The adsorbed particles should form alternating tori or spirals with the thickness determined by the thickness of these layers. Because of different thickness of the chains of the particles for different forms of the competing interactions, the radius of the adsorbing sphere should be properly rescaled to obtain the same patterns as shown in Figure 14 and Figure 15 for 4 and 5 adsorbed tori. We expect that the geometrical procedure with the alternating two ’ropes’ that are wrapped on the sphere can lead to all patterns made by the adsorbed particles, when the diameter of the ropes is adjusted to the thicknes of the self-assembled chains of the particles. We should stress that in addition to the pattens shown in Figure 14 and Figure 15, also patterns corresponding to different ratios of the radius of the adsorbing sphere and the thickness of the chain of the particles can be predicted by our geometrical procedure.

Let us finally mention that in addition to the interesting patterns self-assembled in shells with spherical or elipsoidal boundaries [38,39], different new shapes and new distribution of the aggregates such as micelles or clusters may appear inside slits, cylinders, spheres, or branched channels [38,39,40,41,42,43,44,45,46,47,48], but mixtures of self-assembling nanopaticles in such confinement have not been studied yet.

## 5. Conclusions

We found that a colloidal particle placed in solvent containing a dilute mixture of nanoparticles with competing interactions can be covered by a thick layer of nanoparticles with a new, nontrivial structure. The distribution of the nanoparticles in the adsorbed layer depends on the size of the colloidal particle and on its interactions with the nanoparticles, and variety of different patterns associated with different thickness of the adsorbed layer can be formed.

In our generic model, alternating layers of the two types of nanoparticles are energetically favored. When both components are strongly attracted to the colloidal particle, then the adsorbed nanoparticles self-assemble into alternating parallel tori around the adsorbing sphere, and clusters at its poles (Figure 4). In addition to this structure, the chains of the particles can form alternating spirals wrapped around the colloidal particle (Figure 5 and Figure 6).

Interestingly, the spiral patterns can be obtained from the tori with the caps at the poles of the large sphere by a simple geometrical procedure described in Section 4. With the same procedure, all patterns self-assembled in the one-component SALR system inside a spherical shell with hard walls were predicted. Since this method works well for different systems in different conditions, it is plausible that the long-lived patterns in various systems self-assembling into alternating stripes on a spherical adsorbing surface can be predicted by the geometric construction decribed in Section 4.

When the nanoparticles of the first component are attracted to the colloidal particle and those of the other component are repelled from it, the patterns self-assembled in the adsorbed film are significantly different. For relatively weak attraction of the first component, the adsorbed film is much less ordered. Only for large *R* well-developed alternating stripes are formed. When the first-component particles are strongly attracted to the colloidal particle, however, a smooth, compact monolayer of the attracted particles is formed at the surface of the colloidal particle and screens the repulsion between the colloidal particle and the particles of the second component. The monolayer at the surface is separated from a spherical bilayer of the repelled component by a depletion zone. The subsequent alternating layers of the two components form together a thick layer around the colloidal particle. Although the outer part of the adsorbed layer is porous, the adsorption is significantly larger than in the case of both components attracted to the colloidal particle with the same strength. Moreover, the adsorbed film contains almost the same number of nanoparticles of both components. For different forms of the competing effective interactions between the particles, the alternating spherical concentric layers surrounding the central sphere may have a different thickness. This expectation should be verified for different forms of the competing interactions in future simulations going beyond the scope of this work.

## Data Availability

The data supporting the conclusions of this article will be made available by the authors on reasonable request.
